# Role of Human-Mediated Dispersal in the Spread of the Pinewood Nematode in China

**DOI:** 10.1371/journal.pone.0004646

**Published:** 2009-02-27

**Authors:** Christelle Robinet, Alain Roques, Hongyang Pan, Guofei Fang, Jianren Ye, Yanzhuo Zhang, Jianghua Sun

**Affiliations:** 1 INRA, UR 633 Zoologie Forestière, Orléans, France; 2 The General Forest Pest Control Station, State Forestry Administration, Shenyang, China; 3 Nanjing Forestry University, Nanjing, China; 4 State Key Laboratory of Integrated Management of Pest Insects and Rodents, Institute of Zoology, Chinese Academy of Sciences, Beijing, China; University of Bristol, United Kingdom

## Abstract

**Background:**

Intensification of world trade is responsible for an increase in the number of alien species introductions. Human-mediated dispersal promotes not only introductions but also expansion of the species distribution via long-distance dispersal. Thus, understanding the role of anthropogenic pathways in the spread of invading species has become one of the most important challenges nowadays.

**Methodology/Principal Findings:**

We analysed the invasion pattern of the pinewood nematode in China based on invasion data from 1982 to 2005 and monitoring data on 7 locations over 15 years. Short distance spread mediated by long-horned beetles was estimated at 7.5 km per year. Infested sites located further away represented more than 90% of observations and the mean long distance spread was estimated at 111–339 km. Railways, river ports, and lakes had significant effects on the spread pattern. Human population density levels explained 87% of the variation in the invasion probability (*P*<0.05). Since 2001, the number of new records of the nematode was multiplied by a factor of 5 and the spread distance by a factor of 2. We combined a diffusion model to describe the short distance spread with a stochastic, individual based model to describe the long distance jumps. This combined model generated an error of only 13% when used to predict the presence of the nematode. Under two climate scenarios (stable climate or moderate warming), projections of the invasion probability suggest that this pest could expand its distribution 40–55% by 2025.

**Conclusions/Significance:**

This study provides evidence that human-induced dispersal plays a fundamental role in the spread of the pinewood nematode, and appropriate control measures should be taken to stop or slow its expansion. This model can be applied to Europe, where the nematode had been introduced later, and is currently expanding its distribution. Similar models could also be derived for other species that could be accidentally transported by humans.

## Introduction

Intensification of world trade is a major factor explaining the increase in the number of exotic species introduced everywhere in the world [1]. Invertebrates, mostly insects and nematodes, represent the greatest proportion (∼90%) of the organisms intercepted every year in Europe [2]–[3]. Insects are also the dominant intercepted organisms entering the United-States, representing 73–84% of the total [4]. Invasions depend on biological and climatic factors, but another important factor that has been mostly neglected until now is the economic factor [5]. In China, the rapid increase in the number of introduced species since the 1970s is most likely due to economic growth first, and then, to a lesser extent, climatic factors [5]. While only a small fraction of these biological invaders are able to survive and establish a new population [6], some of them cause a dramatic impact in the newly invaded environment, resulting in great ecological damage and economic loss [7]–[8]. Understanding invasion processes for assessing the risk of biological invasions and improving the management of invasive species has thus become a major challenge [9]–[10].

In this paper, we will deal with the invasion processes of an insect-vectored nematode in China. This nematode constitutes a model organism because: (1) it is spreading very rapidly throughout China, largely affecting tree survival, (2) Chinese territory is large enough to study long distance dispersal, and (3) China is a hotspot of international trade since it entered the World Trade Organization on December 11, 2001.

The pinewood nematode, *Bursaphelenchus xylophilus* (Steiner & Buhrer) Nickle, is native to North America [11]. Since the beginning of the 20^th^ century, it has been accidentally introduced into several Asian countries (Japan- 1905, China- 1982, Taiwan- 1985, Korea- 1988). Then, it reached Europe where it was discovered in Portugal in 1999 [12]–[13]. Despite an intensive containment program in the Setúbal Peninsula where it was restricted since its arrival, the pinewood nematode is at present expanding to other parts of Portugal [14]–[16]).

In China, the nematode was discovered for the first time in Nanjing in 1982. Another infested area was found at more than 1000 km away from the previous one, near Hong-Kong, in 1988, supposedly resulting from a separate introduction. Genetic diversity and phylogenetic analysis suggest that, with the exception of populations in Mingguang, nematode infestations probably originated from Nanjing [17]. The pinewood nematode is vectored by long-horned beetles, mainly the Japanese pine sawyer, *Monochamus alternatus* Hope (Coleoptera: Cerambycidae). Almost 70 tree species have been listed as susceptible to the pinewood nematode but *Pinus* spp. constitute the most susceptible host plants [18]–[23]. Some of these pine species, especially the most affected one, Masson pine (*P. massoniana*), are widely distributed throughout China [24].

Ecophysiological models have already been developed to describe the host-vector interaction and the spread of the nematode among pine stands in Japan [25]–[27]. These models accurately describe the transmission of the disease based on empirical data. Long distance dispersal has been considered in terms of the proportion of beetles with the greatest dispersal capabilities (average long distance dispersal: 1.8 km [26]). However, there was no research describing the effects of human-mediated dispersal at larger scales until now.

Two measures can be used to calculate a confidence interval of the dispersal rate: (1) the distance from the nearest neighbour, and (2) the distance from the introduction point (see [28]–[29] for the dispersal rate of Argentine ants). When individuals (from plant or animal species) disperse randomly, reaction-diffusion models are generally used to describe the natural expansion of the populations (e.g. [30]–[33]). Their mathematical properties are well-known [32], [34]–[35]: they can generate a travelling wave with a constant asymptotic speed 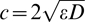, with *D* the diffusion coefficient and *ε* the growth rate [35]–[36].

Human activities, such as logging or trade which requires wooden packaging material, increase the risk for accidental transportation of infested materials and may be responsible for the rapid spread of the nematode. Therefore any connection between infested and non-infested areas (e.g., via highways, railways, rivers, or an electric power network with wooden poles) probably increases the risk of invasion (GF Fang, pers. com.). Transportation hubs have been reported to play an important role in occasional long-distance, human mediated dispersal [37]. Thus, we also investigated the role of anthropogenic pathways and we developed a model combining short- and long-distance spread to gain a greater understanding of the dispersal mechanism and predict any future range expansion.

Human population density could explain a large part of long-distance dispersal for some insect species, as has been shown for the horse chestnut leafminer, *Cameraria ohridella* Deschka & Dimic, in Europe [38]–[39], and the emerald ash borer, *Agrilus planipennis* Fairmaire, in North America [40]. Because the movements of people, cars and trucks also increase the probability of moving infected beetles or infested wood, we carefully investigated the effect of the human population density as an indicator of human-mediated dispersal risk.

Many studies on the climatic tolerance of the carrier beetle have been conducted in Japan and China. Two main thresholds associated with the beetle survival and distribution have been determined: (1) the mean air temperature in July should be above 21.3°C, and (2) the mean temperature in January should be above −10°C [41]–[42]. Moreover, the pinewilt disease has never occurred in North America or Japan when the mean air temperature of the warmest month is lower than 20°C [11]. Territorial expansion of alien species often results not only from human-mediated dispersal but also from climate change [43]. The distribution of many plant and animal species is already affected by climate change (e.g. [44]–[47]). A temperature rise of 2°C could considerably increase the risk of the pine wilt disease development in north western Spain [48]. Consequently, we tested the potential effects of a temperature increase on the spread of this nematode in China.

## Methods

### Data description

#### Pinewood nematode

**Figure 1:**
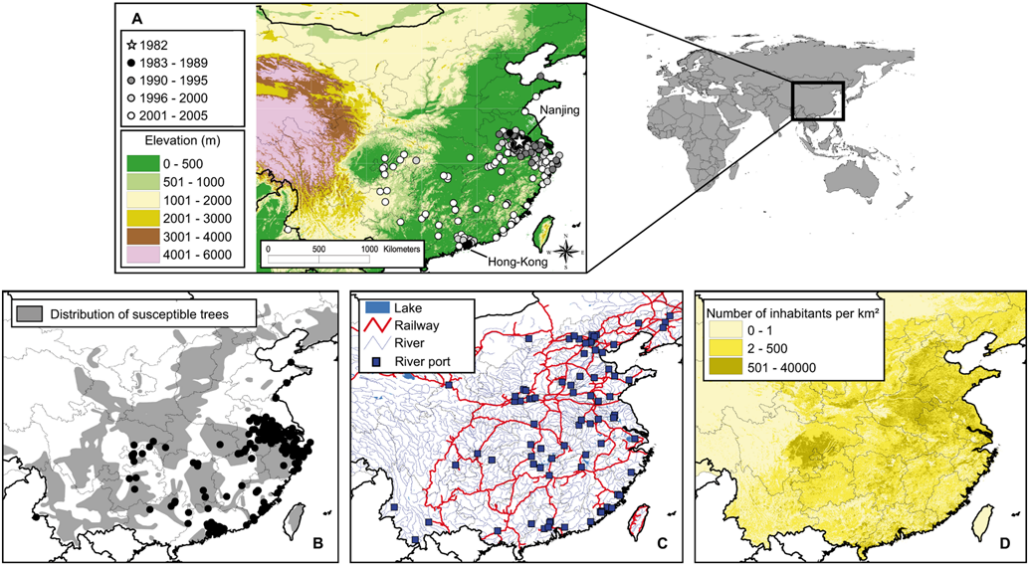
Invasion data and potential driving factors. The pinewood nematode invasion in China between 1982 and 2005 (Panel A). The first pinewood nematode observation in China (in Nanjing) is represented by a white star. Spatial distribution of 10 susceptible tree species (Panel B). Black dots represent locations already infested. Potential anthropogenic pathways (Panel C), and spatial distribution of human-population density in 2000 (Panel D).

**Figure 2:**
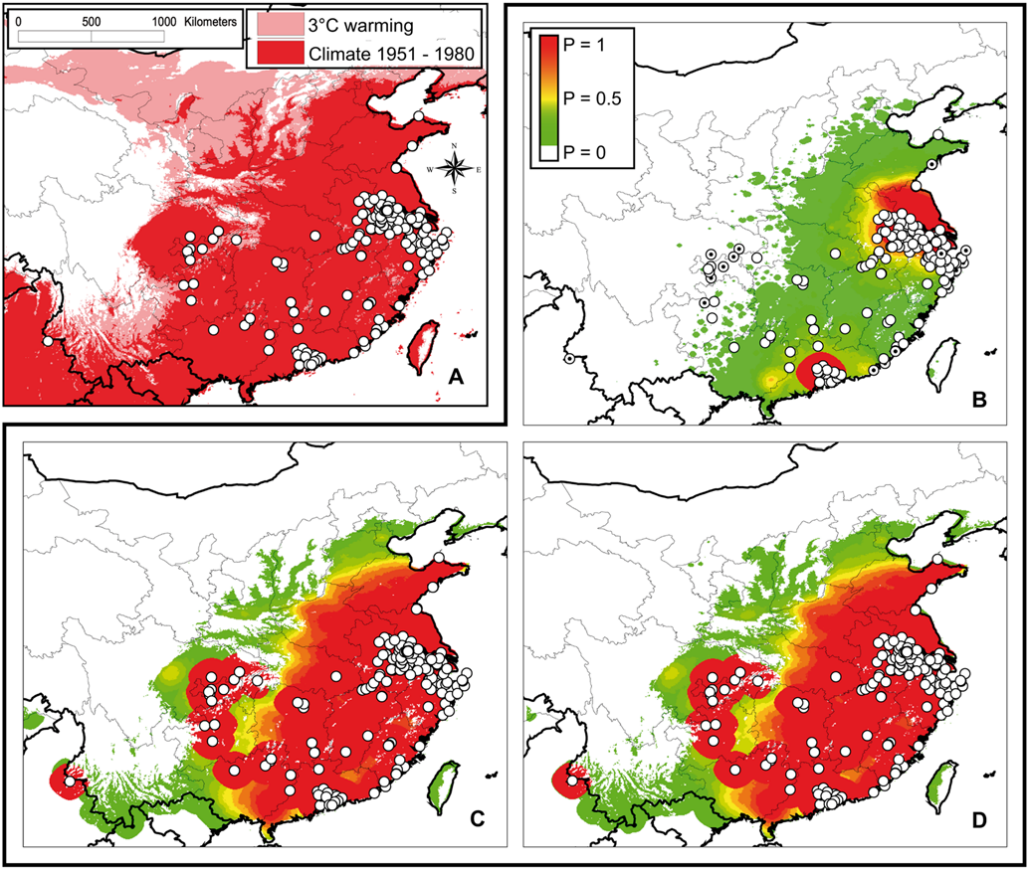
Climate suitability and model prediction of the invasion probability under various climate scenarios. Potentially favourable areas according to normal temperatures over 1951–1980 (dark red), and 3°C of temperature increase (dark+light red is the potential expansion area) (Panel A). White dots represent locations already infested. Invasion probability predicted by the dispersal model: in 2005 (Panel B), in 2025 under the assumption of a stable climate (Panel C), and in 2025 under the assumption of a constant warming (+0.03°C/yr) (Panel D). White dots represent locations infested until 2005, and in Panel A, white dots with a black point inside represent infested locations where the predicted invasion probability in 2005 is zero.

Data was collected by the General Forest Pest Control Station (Shenyang, China), the administration in charge of managing invasive species in China. Two data sets were analyzed: (1) a history of invasion between 1982 and 2005: a list of 171 locations invaded by the pinewood nematode and the year of the initial observation for each location (Fig. 1A), and (2) a monitoring data set summarizing the number of dead trees attributed to the pinewood nematode over 15 years at 7 locations (Table 1). Random samples were collected with Baermann funnels and the samples were examined microscopically. We attributed the death of a tree to the pinewood nematode according to the following typical symptoms: browning needles, many sawyer oviposition scars, declining of resin excretion, and blue xylem stains.

**Table 1:** Monitoring data set.

Location and year of first observation	Mingguang City (1988)	Dangtu County (1992)	He County (1988)	Xiangshan County (1991)	Dinghai District (1992)	Jurong County (1987)	Runzhou District (1988)
Year after first observation							
1	114	165	83	128668	48893	2201	2500
2	23	19130	2000	36600	252771	3936	3970
3	3438	29462	850	15800	227846	12334	4850
4	1432	113000	2560	11550	659537	5700	5320
5	8419	21310	2363	45017	835518	18530	8940
6	2744	18301	1280	149590	851355	14655	12861
7	5796	30862	730	310886	1508139	12667	36754
8	8188	31518	5840	2113538	836800	13246	4177
9	4484	32498	15254	2368897	798324	33876	17409
10	7759	19019	9000	1629464	477020	40610	13876
11	18300	15423	570	642774	167045	35239	15805
12	14004	13707	5020	299408	59100	45187	13413
13	9821	9163	4742	223047	31980	79104	16310
14	154	2503	3612	195873	15806.00	76143	13650
15	3108	1802	3612	119453	8417	76834	7760

Annual changes in the number of dead trees over fifteen years after the introduction of *Bursaphelencus xylophilus* at seven locations in China.

#### Host trees

We considered the 10 native susceptible species with the largest distributions in China (Fig. 1B [24], [49]–[50]): *Pinus armandii* Franch, *Pinus bungeana* Zucc. ex Endl., *Pinus densiflora* Sieb. & Zucc., *Pinus kesiya* Royle ex Gordon var. *langbianensis* (A. Chev.) Gaussen ex N.-S. Bui, *Pinus koraiensis* Sieb. & Zucc., *Pinus massoniana* Lamb., *Pinus sylvestris* L. var. *mongolica* Litv., *Pinus tabuliformis* Carr., *Pinus taiwanensis* Hayata, and *Pinus yunnanensis* Franch. We used GIS maps to represent the ranges of these susceptible tree species. The maps were provided by the Institute of Geographic Sciences and Natural Resources Research (Chinese Academy of Sciences, 11A Datun Road, Chaoyang District, Beijing 100101, People's Republic of China).

#### Potential anthropogenic pathways and human-population density

To investigate the role of anthropogenic pathways, our study utilized the GIS maps of rivers, river ports, lakes, railways (Fig. 1C), and the spatial distribution of human-population density in 2000 (Fig. 1D). These data were also provided by the Institute of Geographic Sciences and Natural Resources Research (Beijing).

### Data analysis and models

#### Climate suitability

The mean temperature in January (*TJan*) and the mean temperature in July (*TJul*) were estimated from a multiple regression using latitude, longitude and elevation as predictive variables, and the data of 279 weather stations in China, over 1951–1980, were used to fit the regression model [51] (data were provided by the Department of Geography, Mainz University, Germany, http://www.webgis-china.de/). Then, these temperatures were interpolated on a grid cell (720×600 cells, with a cell size Δ*x* = Δ*y* = 0.04°, also used hereafter for simulations of the model) covering our study area (20°00–45°00 N, 95°00–125°00 E). For this purpose, we used a digital elevation model derived from the USGS/NASA SRTM data [52]. Based on the developmental thresholds previously defined for the carrier beetle, we determined the areas where the climate was suitable (*TJul*≥21.3°C and *TJan*≥−10°C) and we assumed that, once arrived at a given location, the pinewood nematode could establish only if the climate was suitable, otherwise the invasion would fail. To explore the effects of global warming, we also tested the effects of a temperature increase (see the model description for further details).

#### Short-distance dispersal model

We developed a linear regression between the distance spread and years using the invasion dataset from the area of the first introduction point in China (up to 100 km from Nanjing). To assess short-distance dispersal ability, we considered the spread from this single point because, in other locations, migrants could come from many sources and it was not possible to distinguish short and long distance dispersal. Then, we applied a reaction-diffusion model to describe this short-distance dispersal:
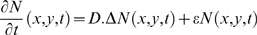
(1)where *N* is the population density of the nematode being carried by the beetles, *t* the time, *x* and *y* the geographic coordinates, *D* the diffusion coefficient and *ε* the growth rate.

To estimate the parameter *ε*, we calculated the growth rate for each year, at the 7 locations using the monitoring data set. For this, we assumed that the ratio of dead trees was a good indicator of the pinewood nematode growth rate [53]. Thus, we calculated *ε*_i_ = ln( *T_i_* / *T_i–1_*), where *T_i_* is the number of dead trees in year *i*, at a given location. Based on this value and the observed rate of population expansion, we determined the value of the diffusion coefficient *D* using the formula 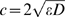.

#### Long-distance dispersal model

Since the source of the new populations was uncertain, we identified the points which could potentially be invaded via short-distance spread. They were located at a distance that could be traversed in one generation (i.e. one year) from previously infested areas. That distance was estimated from the observed expansion rate at one of the original infestation areas near Nanjing. We thus quantified the relative importance of short-distance and long-distance dispersal. Then, to analyze long-distance dispersal in-depth, we used two methods to estimate the distance spread: (1) distance from the nearest neighbour, and (2) distance from the introduction point. Since there were probably two introduction points (in Nanjing area in 1982, and in Hong-Kong area in 1988), we calculated the distance from both introduction points (if the area was infested after 1988) and selected the shortest distance. For the locations infested before 1988, we considered only the distance from the first introduction point in Nanjing area.

Then, we investigated the effects of potential anthropogenic pathways such as rivers, river ports, lakes, railways and the human population density on the long-distance dispersal pattern. Since it was difficult to determine the appropriate spatial scale to detect the effects of these pathways, we considered three neighbourhoods around each invasion point: *N*120, a grid cell composed of 121×121 cells (60 cells on the left, on the right, above and below the invasion point, representing ca. 245.10^3^ km^2^), *N*60, a grid cell composed of 61×61 cells (30 cells in each direction, representing ca. 62.10^3^ km^2^), and *N*30, a grid cell composed of 16×16 cells (15 cells in each direction, representing ca. 16.10^3^ km^2^). We calculated the proportion of cells occupied by each pathway (river, railway and lake), the number of ports and the mean human population density in these neighbourhoods. We made these calculations using the invasion dataset and also a second dataset (of the same size, *n* = 156) selected randomly over China in order to test whether the spread patterns were significantly different from what we would expect at random. We logarithmically transformed these numbers and proportions, and used the Jarque Bera test [54] to check whether the data were normally distributed. Since most of them were not normally distributed, we used a non parametric test (Wilcoxon test) to compare both datasets (observations and randomly generated dataset).

We also calculated the correlation between spread distance and the human population density in the infested area, and we calculated the invasion probability as a function of the human population density. Since the human population is heterogeneously distributed over China, we calculated a corrected invasion probability taking into account the frequency of human densities as follows: (1) we counted the number of infested areas in which the human population density was in the interval [2500 *n*, 2500 *n*+2500[, for *n* = 0, …, 15; (2) we counted the number of cells (from the grid cell used for interpolating temperature data) in which the human population in China was in the same interval; (3) we divided the first number by the second, and expressed it finally as a proportion. Thus, the probability of the invasion of a given area was adjusted with regard to the frequency distribution of human population density in China.

To define the long-distance dispersal kernel, the previous probability (associated with the human population density) was multiplied by the probability to disperse at a given distance. Since long-distance dispersal was noticeably different before and after 2001, we applied a Gaussian kernel with different parameters. These parameters were estimated using the least-squares method.

#### Combination of short and long distance dispersal models

To explicitly model the pinewood nematode expansion, short- and long-distance spread models were combined. Based on the infestation pattern around Nanjing, we determined the number of years, *N_D_*, an area should be infested before it can provide long-distance dispersers and we included this delay in the model.

Invasion of the Hong-Kong area was first observed in 1988 with 6 infested locations, therefore the initial introduction probably took place much earlier. In 1988, infested sites were concentrated in a 27.3 km diameter region. Based on both the observed short-distance dispersal rate of the beetles (estimated around Nanjing) and the infested area around Hong-Kong reported in 1988, we estimated the year of first introduction around Hong-Kong.

A diffusion model (Eq. 1) was applied to describe short distance dispersal, with parameters *D* and *ε* estimated in a previous section. The population density was rounded to 0 in areas where climate was not suitable. The cell was considered infested when the population density reached 1.

Then, we randomly chose the number of long distance dispersers using a normal distribution with the previously determined mean and standard deviation. Parameters of this normal law were different before and after 2001. In reality, even if the main pattern is characterized by a sudden increase of invasions since 2001, the number of long distance dispersers would probably increase with the number of infested sites. Though, to avoid longer computation time, we assumed that the number of dispersers was independent of the number of infested sites.

For each disperser, we randomly chose a cell, infested for at least 6 years, from which it could emigrate, and we randomly selected the cell in which the disperser can settle following the long-distance dispersal kernel (Gaussian kernel multiplied by the probability to invade a location given the human population density, and then normalized). If the climate was not suitable in this cell, we assumed that this invasion failed. Distribution of host trees was not considered in the model because locations of infested sites were not correlated with host tree distribution.

To test the effects of an increase in temperature on the nematode spread, we made two types of simulations: (1) temperatures remain constant ( = mean temperatures over 1951–1980), and (2) temperatures increase linearly. According to climate model projections, global surface temperature will probably rise by 1.1°C–6.4°C between 1980–1999 and 2090–2099 [55]. Consequently, we tested a linear warming of 0.03°C per year, which is close to the mean predicted warming. For simplification, no stochasticity was introduced in this climate scenario.

We made 300 replicate simulations from 1982 to 2005, and we calculated the invasion probability in each cell as the number of times the model predicted the infestation of the cell, divided by 300. The spatial distribution of the invasion probability was compared to the pinewood nematode observations in 2005 to evaluate the fitting success of the model. An independent dataset is generally required to assess the model performance, but due to the complex dispersal processes, it was not possible to select a limited subset of this data and use the model to produce meaningful results. To complete this study, a projection for 2025 was calculated, with and without a temperature increase, using the infested locations (observations) up to 2005 as the initial population in the model. We made 300 replicate simulations for each model (with and without climate warming).

All these analyses and simulations were performed using R language [56]. The coastline extractor from the National Geophysical Data Center, NOAA Satellite and Information Service (http://www.ngdc.noaa/ngdc.htlm), provided a convenient file of China's border for the use in R. Maps were generated using ArcView 9.2, ESRI.

## Results

### Analysis of invasion data

The number of newly infested areas per year was significantly greater after 2001 (t test: *t* = 5.7031, df = 4.638, *P* = 0.003) (Fig. 1A). The mean number of newly infested areas was nearly five times the number of pre- 2001 infestations: the mean±SD was 3.89±3.18 before 2001, and 19.40±5.86 after. Of the 171 infested areas, only 93 (54%) were located within the distribution of susceptible trees documented here.

### Climate data

Temperatures in both January and July were weakly correlated (*R* ^2^<0.30). The following regressions explained 96% and 89% of the variation in the mean temperature in January (*TJan*) and the mean temperature in July (*TJul*), respectively:
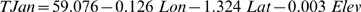
(2)

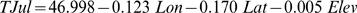
(3)where *Lon* is longitude (decimal degrees), *Lat* is latitude (decimal degrees) and *Elev* is elevation (m). All *P*-values were highly significant (*F*_3,273_ = 2100, *P*<0.001 for Eq.2, and *F*_3,273_ = 723.3, *P*<0.001 for Eq. 3).

Suitable climate area was consistent with the pinewood nematode observations (Fig. 2A). A large part in the south-eastern China is favourable for the nematode establishment. With a 3°C warming, the suitable area could expand by 40%, especially in the northern and north-western regions (Fig. 2A), but the Tibetan Plateau and the surrounding mountainous area, and also the northern part of Yunnan and the western part of Sichuan would remain quite unfavourable.

### Short-distance dispersal model

**Figure 3:**
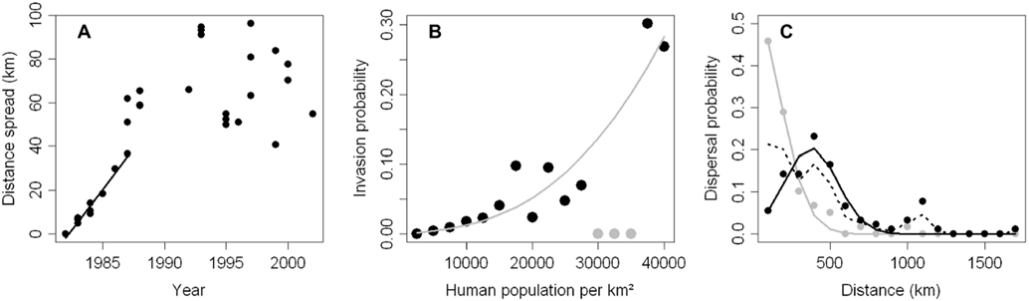
Estimation of short and long distance dispersal ability from data analysis. Distance spread (up to 100 km) from the first introduction point around Nanjing (Panel A). Corrected invasion probability as a function of human population density (Panel B). The grey line is the regression line. Grey dots were discarded from this analysis. Long-distance dispersal kernels (Panel C): observed probabilities over 1982–2005 (dash line), observed probabilities before and after 2001 (grey and black dots, respectively), and estimated probabilities before and after 2001 (grey and black lines, respectively).

The analysis of the distance spread from the first introduction point with years revealed different patterns before and after 1987 (Fig. 3A). Indeed, the relationship between distance and years was linear during the first few years (Pearson's correlation = 0.98; t test: *t* = 15.2899, df = 8, *P*<0.001), while there was no such relationship later (Pearson's correlation = 0.10; t test: *t* = 0.4405, df = 19, *P* = 0.665). Between 1982 and 1987, the pinewood nematode invaded surrounding areas at a constant speed: *c* = 7.5 km/year. Furthermore, the range of maximum growth rates was *ε* = 0.51–2.17. When we considered the maximum value, the net reproductive rate was exp(*ε*) = 8.76. Using the formula 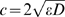 with *ε* = 2.17 and *c* = 7.5 km per year, we found *D* = 6.480 km^2^/year, and we used these estimates in the short-distance dispersal model.

### Long-distance dispersal model

After eliminating local spread (spread within 7.5 km), 156 sites of the 171 appeared to result from long-distance dispersal. Thus, short-distance dispersal represented only 8.8% of the pinewood nematode dispersal. The mean number of long distance dispersals per year was close to the total number of dispersals, the mean±SD was 3.41±3.27 before 2001, and 18.2±5.89 after. These values were used as parameters of a normal law to estimate the number of long distance dispersers each year.

Estimates of long-distance dispersal were significantly higher with the second method (distance from an introduction point: mean = 338.62 km, SE = 23.85 km, median = 271.23 km, *n* = 150) in comparison with the first method (distance from the nearest neighbour: mean = 111.06 km, SE = 11.68 km, median = 53.83 km, *n* = 150) (paired t-test, log transformed, *t* = 18.73, d.f. = 149, *P*<0.001). Records around Hong-Kong in 1988 (*n* = 6) were excluded from this analysis because they were considered as a secondary introduction from abroad, independent from populations originating from Nanjing.

Using both methods, distance spread before 2001 was significantly lower than after this date (t-test, log-transformed, *t* = −3.80, d.f. = 143.18, *P*<0.001 for method 1, and *t* = −7.84, d.f. = 104.16, *P*<0.001 for method 2; the mean±SE were 73.39±10.38 km before 2001 and 135.48±12.09 km after, for method 1; and 171.17±13.46 km before 2001 and 447.18±24.96 km after, for method 2). The mean distance spread approximately doubled.

Effects of rivers were not significant whereas railways, ports, lakes and human population densities had a significant effect on the spread pattern (Wilcoxon test, *P*<0.001). Infested areas were more likely to be located nearby these pathways than points chosen at random (Table 2). The median human population density in areas where the pinewood nematode was present was three times larger than the median human population density in areas selected at random (ca. 3000 inhabitants per km^2^ vs 1000; Table 2).

**Table 2:** Pathways.

		Rivers	Railways	Ports	Lakes	*H*
*N*120	*W*	12261	13381	17345	24336	81
	*p value*	0.908	0.128	<0.001	<0.001	0.956
	*Median* (obs.)	0.160	0.038	5.5	0.026	2853.8
	*Median* (rand.)	0.148	0.034	2	0.004	1168.2
*N*60	*W*	11800	14881	16379	16706	20320
	*p value*	0.645	<0.001	<0.001	<0.001	<0.001
	*Median* (obs.)	0.160	0.046	1	0.029	2935.3
	*Median* (rand.)	0.157	0.033	0	0.002	1169.6
*N*30	*W*	12164	16069	15088	15835	20354
	*p value*	0.997	<0.001	<0.001	<0.001	<0.001
	*Median* (obs.)	0.159	0.054	0	0.009	3074.4
	*Median* (rand.)	0.161	0.032	0	0	1019.2

Results of the Wilcoxon test to compare the effects of rivers, river ports, lakes, railways and human population density (*H*) between infested points and random points, for three neighbourhoods (*N*120, *N*60, *N*30). The sample size of both datasets (observed and random data) was *n* = 156.

No direct relationship was observed between the spread distance and the human population density. Spread distance was not correlated directly with the human population density, neither before 2001 (t-test, log-transformed, *t* = −1.22, d.f. = 57, *P* = 0.227 for method 1, and *t* = 0.03, d.f. = 57, *P* = 0.98 for method 2) nor after 2001 (t-test, log-transformed, *t* = 0.37, d.f. = 89, *P* = 0.71 for method 1, and t = 0.76, d.f. = 89, *P* = 0.45 for method 2). However, the probability that the pinewood nematode would invade a given area increased with the human population density when we introduced a correction related to the frequency of human population and discarded outliers (three points with a null invasion probability at more than 30000 inhabitants per km^2^) (Fig. 3B). Human population density (*H*) explained 87% of the variation in invasion probability (*IP*):
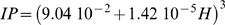
(4)The *P*-value was highly significant (*F*_1,11_ = 76.83, *P*<0.001).

The following regression explained 99% of the variation in the dispersal probability (*γ_LDD_*) before 2001 (with *m* = 0, *σ* = 252.62) and 88% after 2001 (with *m* = 377.22, *σ* = 240.45) (Fig. 3C):
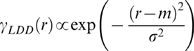
(5)All *P*-values were highly significant (*F*_1,15_ = 1032.8, *P*<0.001 before 2001, and *F*_2,14_ = 42.854, *P*<0.001 after 2001).

### Combination of short and long distance dispersal models

The number of years an area should be infested before it can provide long-distance dispersers was estimated to *N_D_* = 6 years. Based on the invasion pattern near Hong-Kong (6 infested locations discovered in 1988, concentrated in a 27.3 km diameter region), and the results from natural dispersal (7.5 km per year), we estimated that the introduction took place ca. 4 years before, in 1984, and we selected one of the 6 locations to represent the second introduction point in China . As a result, in the model, we considered two introduction points from abroad: Nanjing in 1982 (32°05′ N, 118°48′ E) and around Hong-Kong in 1984 (23°06′ N, 113°26′ E).

The mean invasion probability predicted for locations already infested was 0.59. Only 22 invaded sites (12.9%) were located in a cell where the model predicted an invasion probability in 2005 of 0 (Fig. 2B). They were mainly located in the central part of China and on the coast line. Discarding these points, the mean invasion probability in infested locations was 0.68.

The model predicted an expansion of the potential invasion area (defined by *P*>0) by 47% in 2025 under a stable climate (Fig. 2C), and by 55% under climate warming (Fig. 2D). The invasion probability dramatically increases by 2025, and reached nearly 1 in areas where the invasion probability was predicted to be >0 in 2005. The most susceptible area (defined by *P*>0.95) could expand by 642% in 2025 with a stable climate, and by 662% with climate warming.

## Discussion

The model presented here assesses the role of anthropogenic pathways in the spread of the pinewood nematode. The range expansion from the two source populations, Nanjing and Hong-Kong, was clearly identified, and according to the predicted probability of invasion, the pinewood nematode could become established in all of south-eastern China. The estimated spread rate of the beetle (7.5 km/year) was much higher than that previously observed in Japan (around 2 km/ year [57]–[58], and 4.2 km/year [26]). The net reproductive rate for the carrier beetles (exp(*ε*) = 8.76) was roughly of the same order than previously reported ( = 10.7 [27]).

The method used to determine an annual spread rate did not give accurate long distance estimates but, instead provided a reliable confidence interval (the minimum estimates resulting from method 1 and the maximum estimates resulting method 2). Our analysis revealed a clear difference in the invasion patterns before and after 2001. This breaking point coincided with the entry of China into the World Trade Organization. Although there is no evidence of cause-effect relationships, international trade has probably enhanced internal trade and wood exchange within China. In addition, survey techniques for monitoring pest species have been improved considerably in recent years (e.g. the Global Positioning System), and innovative methods have been developed to reliably detect new pinewood nematode infestations [59]. Improved methods of detection and monitoring may explain why many more infested areas have been discovered since 2000.

Errors in the model projections can be divided into three types: (1) errors in the accuracy of geo-referencing infested areas as well as the precise location of coast lines. These errors should diminish with improvements in geo-referencing methods and technology. (2) Errors resulting from undetected nematode introductions whether they originate as a result of maritime trade or from incursions from neighbouring countries like Myanmar. This problem points out the need for monitoring pest species on a global scale and the exchange of data among countries. (3) Errors in central China where the choice of dispersal kernels is compromised by anthropogenic activity in high population areas. For instance, we should investigate whether the construction of the gigantic Three Gorges Dam (30°49′N; 111°00′E) on the Yangtze River (which flows through Nanjing) has increased the risk of invasion in this central part of China due to intensified transportation of materials.

Climate warming would have little effect on the potential distribution of the pinewood nematode and is negligible when compared to the effects of human-mediated dispersal. In the model, we assume that temperatures will increase linearly and at the same rate throughout China, but important local variability in temperature increase is likely to occur among bioclimatic regions of China [60] (Beijing Climate Center, http://bcc.cma.gov.cn/). This warming was applied to both of the climate variables considered in our model, mean temperature in January (*TJan*) and mean temperature in July (*TJul*) even though winter and summer temperatures may not increase at the same rate [61]. Here we focussed on the effects of an increase in the mean temperature because temperature thresholds had previously been defined [41]–[42]. Other components of climate change will probably affect the pinewood nematode and the carrier beetle. For example, changes in mean precipitation, frequent heat-waves, droughts, floods and storms could potentially play an important role in the population abundance and distribution of many species [62]. Various climatic variables and their combinations should be considered to rigorously assess the effects of climate change on insect populations. Even if all the assumptions we have made are not realistic, they may help to simplify the problem and more detailed models could be developed in the future to more accurately determine the effects of climate change.

Rivers have no apparent effect on nematode spread. The Chinese riparian system is very large and uniformly distributed (Fig. 1C), so it might be difficult to determine its effects using our method. Apart from rivers, other pathways could be involved in the long distance dispersal. The number of human inhabitants is probably a good indicator of the risk for accidental transportation because there is also a good correlation between the human population density and the exchange of wood and wood products [63].

Distribution of susceptible trees cannot, by itself, explain the invasion pattern of the pinewood nematode in China. In this study, we considered the natural distribution of 10 susceptible *Pinus* species which are probably the most widespread host trees. The observed discrepancy between host distribution and infested sites may be the result of nematode colonization of non-native pine plantations and human-made plantations. Their distribution was not included in our analysis. A reliable documentation of this distribution should improve the accuracy of our model predictions. Inclusion of host-tree density data would lead to much better predictions than a simple presence/absence indicator because an invasion may fail due to Allee effects when the density of susceptible trees is too low [25]. Moreover, with better estimates of host-tree density, we may be able to combine our dispersal model with the disease transmission model developed by Yoshimura *et al.* [25]. The resulting model may provide us with more reliable predictions. Because phytosanitary certificates are required for the transfer of wood between provinces, it would be interesting to include these records in our model as an additional variable in the data analysis.

This model was developed to document and quantify the spread of the pinewood nematode spread in China, but it may also have of great potential for predicting the invasion patterns in other areas. In July 1985, the European Plant Protection Organization placed the pinewood nematode on the A1 list of quarantined pests [64]. The risk of invasion of European countries is very high [65] and the disease could become a major threat in European Scots and maritime pine forests [13]. The nematode was discovered for the first time in Portugal in 1999, and containment measures were immediately applied in the Setúbal Peninsula where it was successfully isolated for a few years. Genetic analysis revealed that the nematode found in this region was probably introduced from eastern Asia [66]. Despite governmental actions, the pinewood nematode was recently detected several hundred kilometres from the original infestation source, probably due to human-mediated transportation [16]. A national eradication programme, called PROLUNP, was recently launched for controlling this pest [14], [15]. Consequently, our model could be useful in assessing the invasion risk in Portugal and, at a larger scale, in Europe, and in identifying the most probable invasion pathways so as to improve the management strategies.

Modelling long-distance dispersal is quite difficult due to strong stochasticities, but such predictive models are essential for improving risk assessment and eradication strategies. Gypsy moth, *Lymantria dispar* (L.), is an invasive species which is currently expanding its distribution in North America mainly due to human transportation. Its spread rate was considerably reduced due to the creation of a barrier zone [67]. Eradication strategies and quarantine have also been applied in North America to contain the spread of the emerald ash borer, *Agrilus planipennis* [40]. Our model suggests that the pinewood nematode will probably extend its distribution further into the central and northern provinces of China. Because human mediated dispersal is a fundamental factor in this expansion, authorities should intensively control exchange of wood and wood products between infested and non-infested areas. Various management strategies are available: quarantine to avoid new introductions, eradication to avoid establishment, and a barrier zone to slow the spread [68]. Control measures should be able to break the relationships among the host-tree, the pinewood nematode and the carrier beetle [13], for instance by eliminating or reducing vector beetle populations. Complete eradication of the beetles in a barrier zone is not necessary to control the nematode range expansion because of Allee effects [26]. If the population density is reduced under a certain threshold, then the range expansion speed should decrease and isolated colonies could eventually go extinct naturally.
